# Inference of Cerebrovascular Topology With Geodesic Minimum Spanning Trees

**DOI:** 10.1109/TMI.2018.2860239

**Published:** 2018-07-26

**Authors:** Stefano Moriconi, Maria A. Zuluaga, H. Rolf Jäger, Parashkev Nachev, Sébastien Ourselin, M. Jorge Cardoso

**Affiliations:** 1 CMIC, Translational Imaging GroupUniversity College London 4919 LondonWC1E 6BTU.K.; 2 Facultad deMedicinaUniversidad Nacional de Colombia 28021 Bogotá111321Colombia; 3 Amadeus S.A.S. 83518 06902Sophia AntipolisFrance; 4 Institute of Neurology, University College London 4919 LondonWC1E 6BTU.K.; 5 School of Biomedical Engineering and Imaging SciencesKing’s College London 4616 LondonSE1 7EUU.K.

**Keywords:** Blood vessels, brain, vascular tree, connectivity

## Abstract

A vectorial representation of the vascular network that embodies quantitative features—location, direction, scale, and bifurcations—has many potential cardio- and neuro-vascular applications. We present VTrails, an end-to-end approach to extract geodesic vascular minimum spanning trees from angiographic data by solving a connectivity-optimized anisotropic level-set over a voxel-wise tensor field representing the orientation of the underlying vasculature. Evaluating real and synthetic vascular images, we compare VTrails against the state-of-the-art ridge detectors for tubular structures by assessing the connectedness of the vesselness map and inspecting the synthesized tensor field. The inferred geodesic trees are then quantitatively evaluated within a topologically aware framework, by comparing the proposed method against popular vascular segmentation tool kits on clinical angiographies. VTrails potentials are discussed towards integrating groupwise vascular image analyses. The performance of VTrails demonstrates its versatility and usefulness also for patient-specific applications in interventional neuroradiology and vascular surgery.

## I. Introduction

Vascular image analysis and vessels connectivity are critical to the management of a range of conditions with vast population-level impact [44]. In ordinary clinical practice, the assessment and interpretation of cerebrovascular imaging is hindered on the one hand by the complexity of irreducibly multi-modal 3D scans, and on the other by the pressure of time in the context of rapidly evolving conditions, e.g. mechanical thrombectomy for acute stroke. Moreover, whereas many methods exist for quantifying parenchymal changes (i.e. local vessel morphology, presence of atherosclerotic plaques, surrounding brain lesions), employing *raster* representations of tissue classes, no methods exist for quantifying vascular change where the representations are necessarily *vector*: the geometry of the underlying vascular network. Such vector representations would compactly encode relative, spatial and connectivity-related vascular features, by transcending a predefined and quantized spatial grid, typical of a subject-specific raster angiography [29]. A vectorial representation is not only useful in guiding interventions in individual patients, e.g. guiding intracranial electrode placement [65], catheter motion planning, (un)safe occlusion points identification [15], [40], or endovascular aneurysms repair and stent placement for recanalization [41], [54], but essential for the group-level studies on which both clinical prediction and therapeutic inference ultimately depend. For without a satisfactory means of registering vascular trees across a cohort of patients it is impossible to draw general conclusions about any specific vascular feature. A vectorial representation of the vascular network would therefore allow two forms of *group-level* analysis: *i.* intersubject comparison of geometrical features of the vascular tree (e.g. junction points, branching numbers, tortuousity, and overall haemodynamic properties), and *ii.* intersubject comparisons of various non-vascular parenchymal features, where the brain image-volume is rather registered by its vascular topology [50].

### A. Related Work

Early studies [7], [58], [61] with applications in 3D cerebrovascular image segmentation were first largely concerned with locating a vessel in relation to its neighboring structures, for example to avoid it during neurosurgery or to measure its dimensions at some specific point (e.g. diameter of carotid, level of stenosis or grading of a cerebral aneurysm), where a raster representation is perfectly adequate, and the problem reduces to detecting and voxel-wise segmenting the volume of an object of characteristically local linear morphology. In [35] and [40], a comprehensive collection of methods and techniques for general vascular image segmentation is reviewed, by categorizing different segmentation frameworks by their characteristic strategies (e.g. appearance and geometric models, vascular image features, and extraction scheme). Would these previous studies be motivated and inspired by extracting a descriptive vascular network in the form of a set of connected trees, these seemed to address the problem of vascular connectivity in a rather independent and disjoint manner. Briefly, Frangi *et al.* [25] and Law and Chung [39] first proposed tubular enhancing methods in 3D with the aim of better contrasting vessels over a background. A scale-dependent scalar *vesselness* measure, representing the vascular saliency map, is obtained either by adopting different flavors of the Hessian matrix eigendecomposition, or by determining the image gradient projected on a unit sphere boundary, i.e. the oriented flux. Under the assumption of well-contrasted and locally-linear continuous tubular structures, these methods represent the popular and traditional ridge detectors, however highly tortuous, curvilinear and irregularly shaped tubular structures, together with bifurcating and fragmented vessels with low signal-to-noise ratio (SNR) are often poorly captured [2]. To better detect junction points and trace vascular branches [10], [19], [20] embedded higher-order metrics in a tractography-like framework exploiting vessel anisotropy, directionality and local asymmetry. Tensors were derived either via least-square fitting on the image data by enforcing positive-definiteness, or by combining a scale-dependent metric with the locally optimal vascular orientation. Annunziata *et al.* [1] first introduced a more smoothly connected filtering approach to enhance tortuous tubular structures in 2D, by defining scalar and curvilinear bivariate Gaussian kernels (SCIRD). An extension of the proposed smooth curvilinear filter-bank was presented in [2], where deep learning techniques were combined for boosted performances. As second step, Bullitt *et al.* [15] and Kwitt *et al.* [38] proposed a set of methods to recover a connected network, given a vascular saliency map and a set of initial manually-sampled seeds, or disconnected branches, or fragmented centerlines. Cores were introduced to identify and track bifurcating branches, whereas vascular graphs are recovered using minimum spanning tree algorithms on image-intensity descriptors, or using graph kernels, by matching subtree patterns upon a similarity metric. In [4], a different approach recovers the vasculature *a posteriori*, by determining a set of centerlines as medial axes from the three-dimensional surface model which smoothly segments the lumen of the vessels.

### B. Challenges of Cerebrovascular Topology Inference

To the best of our knowledge, the aforementioned studies mainly addressed the problem of accurately locating and characterizing vessel geometries in a *raster*-like fashion, rather than focusing on the *vectorial* connecting topology. In [24], [56], and [57], the topological reconstruction of connected neighboring structures is traditionally addressed with the extraction of centerlines from a given segmentation by means of a skeletonisation process. Simplicial or cubical complex frameworks [16], [21]–[23] may be required when topological busy junctions are found in 2D or 3D finite raster grids. Alternatively, the skeletonisation is performed with topologically-preserving morphological operators (e.g. erosion and opening). These have been employed also for the design of specific tubular-like ridge detectors [46], [47]. Other formulations [34], [59] extract a $l_{0}$ level-set consisting in minimal paths (i.e. geodesics) to implicitly define connecting branches. In the following, we refer to *vectorial* cerebrovascular topology as the descriptive connectivity and branching pattern of a given set of vascular structures in the brain by adopting a spatially-and connectedness-aware embedding of a graph, which simultaneously encodes geodesics, and transcends a predefined quantized raster grid [29]. In general, the quantitative vectorial description and characterisation of a network become more complex and challenging as the network increases in size and allows for variable connectivity patterns. In our case, the cerebrovascular anatomical intra- and inter-subject variability [31] does not allow for a globally standardized vessel network extraction yet. Malformations and pathologies can also dramatically increase the complexity of the vasculature topology, where a compact representation is sometimes impractical. Complex topologies are required for the characterisation of the whole cerebrovascular system: anastomoses such as the Circle of Willis and those of the capillary bed in the cortex [11], [29] show cyclic connecting patterns at varying scales and depth. In these cases, hierarchical tree-like structures cannot adequately model the underlying anatomy, and a more general and unconstrained graph formulation is required. However, the topological inference of major deep-brain arterial (or venous) vascular trees can be locally projected to multiple-trees extraction strategies. Few topological references and data-driven gold standards of vascular connectivity are available. These, often fragmented or limited to a region of interest, require the thorough annotation of experts at different levels of vascular branching, where minor mis-classifications may significantly affect the topology of the resulting vascular graph [51]. The thorough segmentation of a whole-brain vascular image dataset is considered intractable [12], and it can take up to 8 weeks of manual labor per subject [52]. This constitutes a considerable limitation for any method’s validation. Given the substantial complexity of the task, and the lack of an extensive ground-truth for complete vascular networks, the accurate and exhaustive extraction of the vessel connectivity still remains an open problem.

We present here *VTrails*, a novel method that aims at the fully automatic inference of the vascular network topology, by addressing simultaneously both vessel enhancement and connectivity under a unified consistent mathematical framework. Following the concepts first introduced in [49], the major contributions are:

i definition of a compact Steerable Laplacian of Gaussian Swirls (SLoGS) enhancing filter-bank, representative of local vessel portions at different degrees of tortuosity;
ii description of a multi-resolution, curvilinear- and rotation-invariant filtering framework to simultaneously and consistently synthesize scalar- and tensorial-saliency maps, whose combination yields a smoothly connected Riemannian vesselness potential;
iii design of an adaptive and exhaustive (non-greedy) search of geodesic connecting paths over the Riemannian vesselness potential, determining an over-connected geodesic vascular graph;
iv extraction of the acyclic vascular topology (i.e. the vascular trees) as the minimum spanning trees of the over-connected geodesic graph underlying anatomically correct vascular trees.

VTrails is first described in detail in Section II. The validating experimental set-up, comprising both synthetic and real clinical images, is presented in Section III. Results, reported in Section IV, are evaluated with the available ground-truth; observations and conclusions are drawn for the considered experiments with regards to the geometry of the reconstructed vascular trees and the topological connectivity in Section V. Implementation details and performance benchmarking are listed in the Appendix.

## II. Methods

Aiming at the connectivity enhancement of fragmented, bifurcating and tortuous vessels, we present a multi-resolution filterbank of Steerable Laplacian of Gaussian Swirls (SLoGS), whose elongated and curvilinear Gaussian kernels recover a smooth, connected and orientation aware Riemannian vesselness map. Also, under the assumption that vessels connect by minimal paths, the vascular over-connected geodesic graph is then determined with an exhaustive connectivity paradigm propagating over the synthesized vesselness map, and the topology of the underlying anatomical vascular trees is lastly inferred as the geodesic minimum spanning tree.

We introduce in Section II-A the SLoGS filterbank. Then, a multiscale image filtering framework is described in Section II-B using SLoGS. The scalar and tensorial vesselness components are integrated over scales in Section II-C. An anisotropic level-set combined with a non-greedy connectivity paradigm is presented in Section II-D to determine the vascular over-connected geodesic graph. Lastly, the extraction and refinement of the vascular minimum spanning trees are described in Section II-E.

### A. SLoGS Curvilinear Filterbank

Considering an image $V: \mathbb {R}^{3} \rightarrow \mathbb {R}$, the respective filter response is obtained as $V^{{\textit {filt}}} \triangleq V \ast K$, for any predefined filtering kernel $K \colon \mathbb {R}^{3} \rightarrow \mathbb {R}$. Following the concepts first introduced in [1], [42], and [49] and without losing generality, the SLoGS filtering kernel $K$ is derived here by computing the second-order directional derivative in the gradient direction of a curvilinear Gaussian trivariate function $\Gamma \colon \mathbb {R}^{3} \times \mathbb {R}^{3}_{+} \times \mathbb {R}^{3} \rightarrow \mathbb {R}$. The gradient direction and its perpendicular constitute the first-order gauge coordinates system $(\boldsymbol {\omega },\boldsymbol {\upsilon })$, where $\boldsymbol {\omega } = \frac {\nabla \Gamma }{\| \nabla \Gamma \|} $, and $\boldsymbol {\upsilon } = \boldsymbol {\omega }_{\perp }$, with the spatial gradient $\nabla $. The function $\Gamma $ has the form

\begin{align*} \Gamma \left ({\mathbf {x},\boldsymbol {\sigma },\mathbf {c}}\right) \propto \prod _{d=1}^{3} \frac { 1 }{\sqrt { 2\pi \sigma _{d}^{2}} }~ e^{-\frac {\mathcal {X}_{d}^{2}}{2\sigma _{d}^{2}}}~, \text {with}~ \begin{cases} \mathcal {X}_{1} \,=\, x_{1},\qquad \qquad ~\\ \mathcal {X}_{2} \,=\, x_{2} + c_{0}x_{1} + c_{1}x_{1}^{2}, \\ \mathcal {X}_{3} \,=\, x_{3} + c_{2}x_{1}^{3},\qquad \end{cases}\\ {}\tag{1}\end{align*}

where $\mathbf {x} = x_{1}\underline {\text {i}} + x_{2}\underline {\text {j}} + x_{3}\underline {\text {k}}$, with $\{\underline {\text {i}},\underline {\text {j}},\underline {\text {k}}\}$ the Cartesian image reference system, $\boldsymbol {\sigma }$ modulates the cross-sectional profiles and the elongation of the Gaussian spatial distribution, and the factor $\mathbf {c}$ accounts for both planar asymmetry and two levels of curvilinear properties (e.g. bending and tilting), by quadratic- and cubic-wise deforming the support. Given $\boldsymbol {\sigma }$ and $\mathbf {c}$, $\Gamma \left ({\mathbf {x},\boldsymbol {\sigma },\mathbf {c}}\right)$ represents the smooth impulse response of the 3D Gaussian kernel. By operating a directional derivative on $\Gamma $ along $\boldsymbol {\omega }$, i.e. $\mathcal {D}_{\boldsymbol {\omega }}$, we define the SLoGS kernel $K$ as $K = \mathcal {D}_{\boldsymbol {\omega }} \left [{ \mathcal {D}_{\boldsymbol {\omega }} \Gamma }\right] = \mathcal {D}_{\boldsymbol {\omega }} \left [{ \boldsymbol {\omega }^{t} \nabla \Gamma }\right]$, thus being

\begin{equation*} K \triangleq \boldsymbol {\omega }^{t} H\left ({\Gamma }\right) \boldsymbol {\omega }~,~ \text {where}~~ H\left ({\Gamma }\right) = { \left [{ \begin{matrix} \Gamma _{\underline {{\textit {ii}}}} & \Gamma _{\underline {{\textit {ij}}}} & \Gamma _{\underline {{\textit {ik}}}} \\ \Gamma _{\underline {{\textit {ji}}}} & \Gamma _{\underline {{\textit {jj}}}} & \Gamma _{\underline {{\textit {jk}}}} \\ \Gamma _{\underline {{\textit {ki}}}} & \Gamma _{\underline {{\textit {kj}}}} & \Gamma _{\underline {{\textit {kk}}}} \end{matrix} }\right] } \tag{2}\end{equation*}

is the Hessian matrix of the Gaussian distribution $\Gamma $. Since $\Gamma $ is twice continuously differentiable, then $H(\Gamma)$ is well defined. Also, since $H(\Gamma)$ is symmetric, an orthogonal matrix $Q$ exists, so that $H(\Gamma)$ can be diagonalized as $H(\Gamma) = Q\Lambda Q^{-1}$. The eigenvectors $\underline {q}_{l}$ form the columns of $Q$, whereas the eigenvalues $\lambda _{l}$, with $l=1,2,3$, constitute the diagonal elements of $\Lambda $, so that $\Lambda _{ll} = \lambda _{l}$ and $| \lambda _{1} | \leq | \lambda _{2} | \leq | \lambda _{3} |$. For any point $\mathbf {x}$, $K(\mathbf {x})$ can be rewritten as $K(\mathbf {x}) = \boldsymbol {\omega }^{t} \left ({Q \Lambda Q^{-1}}\right) \boldsymbol {\omega }$. Geometrically, the columns of $Q$ represent a rotated orthonormal basis in $\mathbb {R}^{3}$ relative to the image reference system so that $\underline {q}_{l}$ are aligned with the principal directions of $\Gamma $ at any point $\mathbf {x}$. The diagonal matrix $\Lambda $ characterizes the topology of the hypersurface in the neighborhood of $\mathbf {x}$ (e.g. flat area, ridge, valley or saddle point in 2D) and modulates the variation of slopes, since the eigenvalues $\lambda _{l}$ are the second-order derivatives along the principal directions of $\Gamma $. Factorizing $K(\mathbf {x})$, we have: $K(\mathbf {x}) = (\boldsymbol {\omega }^{t} Q) \Lambda (Q^{-1} \boldsymbol {\omega })$; the gradient direction $\boldsymbol {\omega }$ is mapped onto the principal directions of $\Gamma $. Solving (2), we demonstrate $K$ has the form of a 3D Laplacian of Gaussian ($LoG$), as

\begin{align*} K(\mathbf {x})=&G \left [{ \begin{smallmatrix} \Gamma _{\underline {\text {i}}} \\ \Gamma _{\underline {\text {j}}} \\ \Gamma _{\underline {\text {k}}} \end{smallmatrix} }\right]^{t} \overbrace { \underbrace { \left [{ \begin{smallmatrix} q_{11}&q_{21}&q_{31}\\ q_{12}&q_{22}&q_{32}\\ q_{13}&q_{23}&q_{33} \end{smallmatrix} }\right] }_{Q} \underbrace { \left [{ \begin{smallmatrix} \scriptscriptstyle {\lambda _{1}}&\scriptscriptstyle {0}&\scriptscriptstyle {0}\\ \scriptscriptstyle {0}&\scriptscriptstyle {\lambda _{2}}&\scriptscriptstyle {0}\\ \scriptscriptstyle {0}&\scriptscriptstyle {0}&\scriptscriptstyle {\lambda _{3}} \end{smallmatrix} }\right] }_{\Lambda } \underbrace { \left [{ \begin{smallmatrix} q_{11}&q_{12}&q_{13}\\ q_{21}&q_{22}&q_{23}\\ q_{31}&q_{32}&q_{33} \end{smallmatrix} }\right] }_{Q^{-1} = Q^{t}} }^{H(\Gamma)} \left [{ \begin{matrix} \Gamma _{\underline {\text {i}}} \\ \Gamma _{\underline {\text {j}}} \\ \Gamma _{\underline {\text {k}}} \end{matrix} }\right]\\=&{ \sum _{l=1}^{3}\gamma _{l}\lambda _{l} } = \gamma _{1}\frac {\partial ^{2}}{\partial \underline {q}{_{1}}^{2}}\Gamma + \gamma _{2}\frac {\partial ^{2}}{\partial \underline {q}{_{2}}^{2}}\Gamma + \gamma _{3}\frac {\partial ^{2}}{\partial \underline {q}{_{3}}^{2}}\Gamma \\[4pt]&\triangleq \boldsymbol {\gamma }LoG(\Gamma), \tag{3}\end{align*}

where $\gamma _{l} = G \cdot { \left ({\Gamma _{\underline {\text {i}}}q_{l1} + \Gamma _{\underline {\text {j}}}q_{l2} + \Gamma _{\underline {\text {k}}}q_{l3} }\right)^{2} }$ modulate the respective components of the canonical $LoG$ filter oriented along the principal directions of $\Gamma $, and $G = \frac {1}{\Gamma _{\underline {\text {i}}}^{2} + \Gamma _{\underline {\text {j}}}^{2} + \Gamma _{\underline {\text {k}}}^{2}}$. Note that for vanishing spatial gradients, e.g. at $\mathbf {x} = \mathbf {0}$, we have $\gamma _{l}=\frac {1}{3}$. Given any arbitrary orientation $\tilde{\boldsymbol {\omega }}$ as an orthonormal basis similar to $Q$, the arbitrarily defined dictionary of filtering kernels can steer by computing the rotation transform, which maps the integral orientation basis of each Gaussian kernel $\boldsymbol {\Phi }_{\Gamma } = \frac {\int (\Gamma (\mathbf {x}) \cdot Q(\mathbf {x})) d\mathbf {x}}{\| \int (\Gamma (\mathbf {x}) \cdot Q(\mathbf {x})) d\mathbf {x} \|}$ on $\tilde{\boldsymbol {\omega }}$.

Together with the SLoGS kernel $K\vphantom {^{^{^{}}}}$, we introduce the second-moment matrix $T$ associated to the smooth impulse response $\Gamma $ by adopting the ellipsoid model in the continuous neighborhood of $\mathbf {x}$. Thanks to the intrinsic log-concavity of $\Gamma $, a symmetric tensor $T(\mathbf {x})$ is derived from the eigendecomposition of ${H(\tilde {\Gamma })}$, with $\tilde {\Gamma } = -\log (\Gamma)$, as $T(\mathbf {x}) = Q\,\,\Psi \,\,Q^{-1}$, where $\Psi $ is the diagonal matrix of the canonical unit volume ellipsoid

**Fig. 1.**
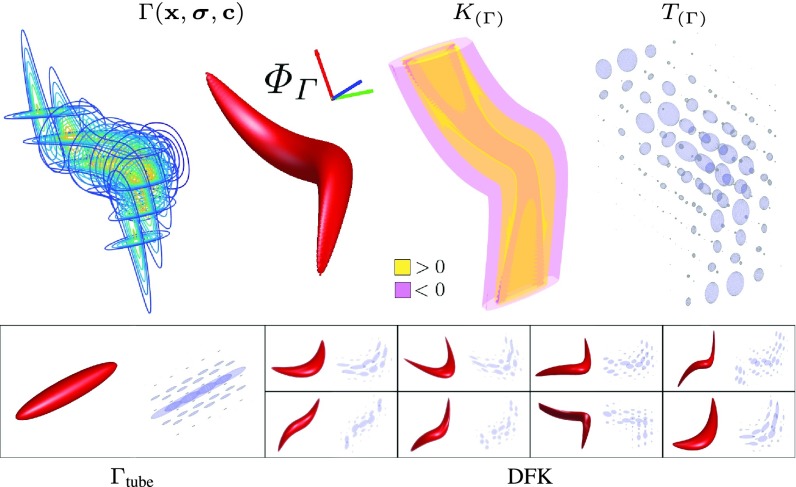
SLoGS filterbank: definition of a dictionary of filtering kernels (DFK).

\begin{equation*} \Psi = \left ({\prod _{l=1}^{3}\psi _{l}}\right)^{-\frac {1}{3}} \cdot \left [{ \begin{smallmatrix} \psi _{1}&0&0\\ 0&\psi _{2}&0\\ 0&0&\psi _{3} \end{smallmatrix} }\right], ~~\text {being}~~ { \left \{{\vphantom {\begin{smallmatrix} \psi _{1} \,=\, \frac {\tilde {\lambda }_{1}}{\sqrt { \tilde {\lambda }_{2}\tilde {\lambda }_{3} }},\\ \psi _{2} \,=\, \frac {\tilde {\lambda }_{2}}{\tilde {\lambda }_{3} },\quad ~\\ \psi _{3} \,=\, 1\qquad \, \end{smallmatrix}} }\right.} \begin{smallmatrix} \psi _{1} \,=\, \frac {\tilde {\lambda }_{1}}{\sqrt { \tilde {\lambda }_{2}\tilde {\lambda }_{3} }},\\ \psi _{2} \,=\, \frac {\tilde {\lambda }_{2}}{\tilde {\lambda }_{3} },\quad ~\\ \psi _{3} \,=\, 1\qquad \, \end{smallmatrix} \tag{4}\end{equation*}

the respective semiaxes’ lengths. The tensor field $T$ is a symmetric positive definite matrix for any $\mathbf {x} \in \mathbb {R}^{3}$, since $\tilde {\Gamma }$ is a convex quadratic form. The manifold of the obtained tensors can be mapped into six independent components in the Log-Euclidean space, which greatly simplifies the computation of Riemannian metrics and statistics [6]. The continuous and spatially smooth tensor field $T$ inherits the steerable property. Resembling diffusion tensor MRI, the SLoGS kernel shows a preferred diffusivity pattern for a given energy potential (e.g. $\Gamma $ in Fig. 1). This allows to eventually determine an arbitrary dictionary of filtering kernels (DFK) which embeds anisotropy and high-order directional features to scalar curvilinear templates, these enhancing and locally resembling typical, smooth vessels. Aiming also at contrasting vascular boundaries and the background component of the image, we similarly introduce an extra pair of degenerate kernels. The pseudo-impulsive $\delta {\textit {LoG}}$ is an isotropic derivative filter given by the $LoG$ of $\Gamma _{\delta }(\mathbf {x},\boldsymbol {\sigma },\mathbf {c} = \mathbf {0})$, representing a Dirac delta function for $\boldsymbol {\sigma }\rightarrow \mathbf {0}$. $\delta {\textit {LoG}}$ is intrinsically sensitive to sharp intensity transitions, capturing therefore edges of vascular structures in angiographic images. The uniformly flat $\nu {\textit {LoG}}$ is the second degenerate Laplacian of Gaussian kernel, which derives from $\Gamma _{\nu }(\mathbf {x},\boldsymbol {\sigma },\mathbf {c} = \mathbf {0})$, assuming a uniform, constant-value for $\boldsymbol {\sigma }\rightarrow \mathbf {\infty }$. Analogously, $\nu {\textit {LoG}}$ is sensitive to regions of homogeneous intensities, capturing thus non-vascular parenchymal structures. Since $\delta {\textit {LoG}}$ and $\nu {\textit {LoG}}$ have singularities and represent isotropic degenerate kernels, we define only their scalar component.

### B. Connected Vesselness Map and the Tensor Field

**Fig. 2.**
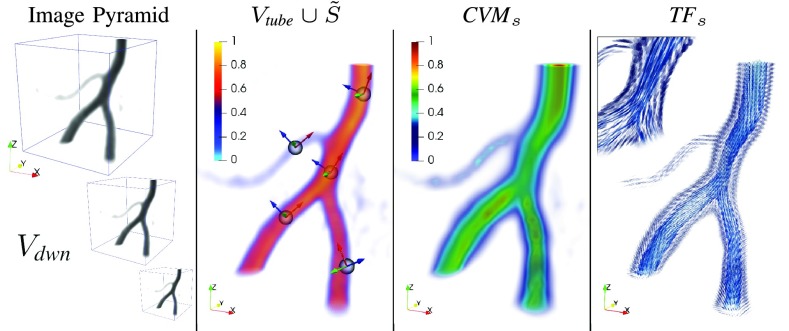
Connected vesselness map and tensor field synthesis at scale $s$.

As recalled in Section II-A and similarly to [26] and [33], the idea is to convolve finite SLoGS kernels with the discrete vascular image in a multi-resolution, curvilinear- and rotation-invariant framework, to obtain simultaneously the scalar connected vesselness map and the associated tensor field. For simplicity and compactness, the multi-resolution filtering will be detailed for a generic scale $s$. Scale-invariance is achieved by keeping the size of the compact-support SLoGS fixed, while the size of the image $V$ varies accordingly with the multi-scale pyramid (Fig. 2). Also, different spatial band-pass frequencies can be modulated with different $\boldsymbol {\sigma }$ of the SLoGS kernels. $V$ is down-sampled firtst at the scale $s$ as in [17] to obtain $V_{{\textit {dwn}}}$. An early tubular saliency map $V_{{\textit {tube}}}$ is then determined as

\begin{equation*} V_{{\textit {tube}}} = { \sum _{\tilde{\boldsymbol {\omega }}_{\text {ico}} \in \Omega _{\text {ico}}} V_{{\textit {tube}}}^{(\tilde{\boldsymbol {\omega }}_{\text {ico}})} }\, \tag{5}\end{equation*}

with $V_{{\textit {tube}}}^{(\tilde{\boldsymbol {\omega }}_{\text {ico}})} = \max \left ({0, V_{{\textit {dwn}}} \ast K_{{\textit {tube}}}^{(\tilde{\boldsymbol {\omega }}_{\text {ico}})} }\right)$. $K_{{\textit {tube}}}$ is derived from the discretized tubular kernel $\Gamma _{{\textit {tube}}}(\mathbf {x}, \sigma _{1}>\sigma _{2}=\sigma _{3},\mathbf {c} = \mathbf {0})$ (Fig. 1), whereas $\tilde{\boldsymbol {\omega }}_{\text {ico}} \in \Omega _{\text {ico}}$ are the orthonormal bases in $\mathbb {R}^{3}$, derived using an icosphere at subdivision level $n = 2$ for the orientation sampling in 3D. $V_{{\textit {tube}}}$ is meant to provide an initial, coarse, although highly-sensitive set of saliency features in $V_{{\textit {dwn}}}$: the vessel *spatial locations* and *principal orientations* (Fig. 2). Identifying such features has two advantages; first it restricts the problem of the rotation-invariant filtering to an optimal complexity in 3D, avoiding unnecessary convolutions; also, a localized subset of vessel samples can be obtained. The vessel spatial locations are mapped as voxel binary seeds $\tilde {S}$, and the associated set of principal orientations $\Theta $ forms a group of orthonormal basis in $\mathbb {R}^{3}$. $\tilde {S}$ are defined as

**Fig. 3.**
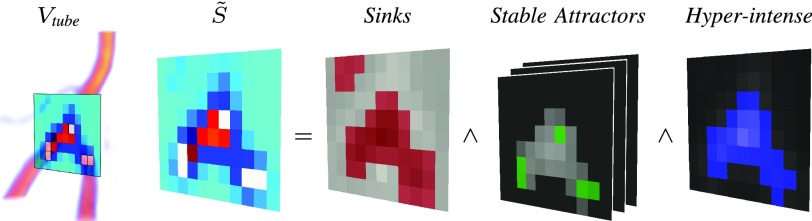
Seeds $\tilde {S}$ detection as in (6). Representative slice in 2D.

\begin{align*} \tilde {S} = \text {div} \left ({\nabla V_{{\textit {tube}}} }\right) < 0~ \wedge ~ \lambda _{1,2,3}^{V_{{\textit {tube}}}} < 0~ \wedge ~ V_{{\textit {tube}}} \geq Q_{p}(V_{{\textit {tube}}}^{+}) \enspace, \\ {}\tag{6}\end{align*}

where $\text {div} \left ({\nabla V_{{\textit {tube}}} }\right)$ is the divergence of $V_{{\textit {tube}}}$’s spatial gradient field, $\lambda _{1,2,3}^{V_{{\textit {tube}}}}$ are the eigenvalue maps derived from the voxel-wise eigendecomposition of $H(V_{{\textit {tube}}})$, and $Q_{p}(V_{{\textit {tube}}}^{+})$ is the percentile of the positive $V_{{\textit {tube}}}$ samples’ pool. Analytically, $\tilde {S}$ represents voxels that concurrently are sinks [58]; that are regarded as stable attracting points of the intensity-based hyper-surface [13]; and that show high-intensities in the tubular saliency map $V_{{\textit {tube}}}$, (Fig. 3). With $\tilde {S}$, the orientations $\Theta $ are automatically determined as the set of eigenvectors associated to $\lambda _{1,2,3}^{V_{{\textit {tube}}}}$ (Fig. 2). The greater the intensity threshold $Q_{p}(V_{{\textit {tube}}}^{+})$, the greater the image noise-floor rejection, the lower the retrieved seeds and the fewer the details detected by $V_{{\textit {tube}}}$. Also, the cardinality of $\tilde {S}$ and $\Theta $ is a trade-off with the convolutional complexity at each scale $s$.

The convolutional analysis/synthesis step can be embedded in a fully parallel filtering framework, by considering the down-sampled image $V_{{\textit {dwn}}}$ and the filtering kernels in DFK, each steered along every principal orientation $\Theta $. The integral connected vesselness map ${\textit {CVM}}_{s}$, at any scale $s$, has the form

\begin{equation*} {\textit {CVM}}_{s} = { \sum _{K \in {\textit {DFK}}} \, \sum _{\boldsymbol {\theta } \in \Theta } V_{\mathcal {S}}^{(K,\boldsymbol {\theta })} }, \tag{7}\end{equation*}

where $V_{\mathcal {S}}^{(K,\boldsymbol {\theta })} = \max \left ({\,0\, V_{{\textit {dwn}}} \ast K^{(\boldsymbol {\theta })} }\right)$ is the filter response given the considered SLoGS kernel (Fig. 2). Similarly, the boundaries and background scalar maps, i.e. ${\textit {BDM}}_{s}$ and ${\textit {BGM}}_{s}$ respectively, are determined at each scale $s$

\begin{align*}& {\textit {BDM}}_{s} = \max (-\infty \,\overline {V}_{{\textit {dwn}}} \ast \delta {\textit {LoG}}), \tag{8}\\&~~ {\textit {BGM}}_{s} = \max (\,0\,\overline {V}_{{\textit {dwn}}} \ast \nu {\textit {LoG}}), \tag{9}\end{align*}

where, in this case, $\overline {V}_{{\textit {dwn}}}$ is the image negative of $V_{{\textit {dwn}}}$.

The anisotropic tensor field ${\textit {TF}}_{s}$ is synthesized and normalized in the Log-Euclidean space as the integral *weighted-sum* of the steered tensor patch associated with the kernel with maximal filter response over $V_{{\textit {dwn}}}$, centered at the voxel $\mathbf {v}$, and has the form (10), as shown at the bottom of the next page,

**Figure:** \begin{equation*} { {\textit {TF}}_{s,({\textit {LE}})} = \frac {1}{W} \sum _{K \in {\textit {DFK}}} \sum _{\boldsymbol {\theta } \in \Theta } \left ({\sum _{\lfloor \mathbf {v} \rceil } \overbrace { V_{\mathcal {S}}^{(\mathbf {v},K,\boldsymbol {\theta })} \cdot \Gamma _{(K)}^{(\boldsymbol {\theta })} \cdot \Xi }^{ \text {weights}} \cdot \overbrace { T^{(\boldsymbol {\theta })}_{K,({\textit {LE}})} }^{ \text {patch}} }\right) },~~\text {with}~~ \begin{cases} \det {\left ({{\textit {TF}}_{s} (\mathbf {v}) }\right)}\,\,=\,\, 1,\,\qquad \qquad \qquad \qquad ~\\ W = \sum _{K \in {\textit {DFK}}} \sum _{\boldsymbol {\theta } \in \Theta } \sum _{\lfloor \mathbf {v} \rceil } V_{\mathcal {S}}^{(\mathbf {v},K,\boldsymbol {\theta })} \cdot \Gamma _{(K)}^{(\boldsymbol {\theta })} \cdot \Xi, \\ \end{cases} \normalsize \tag{10}\end{equation*}

where $W$ is the integral normalizing weight-map accounting for the steered curvilinear kernels; $V_{\mathcal {S}}^{(\mathbf {v},K,\boldsymbol {\theta })}\vphantom {^{^{^{^{^{}}}}}}$ is the modulating SLoGS filter response at $\mathbf {v}$ as in (7); $\Gamma _{(K)}^{(\boldsymbol {\theta })}\vphantom {^{^{^{^{^{}}}}}}$ is the steered Gaussian impulse response associated to the kernel $K$ in DFK; $\Xi $ is the Hann smoothing window in the neighborhood $\lfloor \mathbf {v} \rceil $ centered at $\mathbf {v}$, and $T^{(\boldsymbol {\theta })}_{K,({\textit {LE}})}$ is one of the six independent components of the discrete steered tensors patch $T$ in the Log-Euclidean domain. Note that all 6 tensorial components are equally processed, and that the neighborhood $\lfloor \mathbf {v} \rceil $ and the SLoGS tensors patch $T^{(\boldsymbol {\theta })}_{K,({\textit {LE}})}$ have the same size.

### C. Multi-Scale Maximal Integration

Each scale-dependent contribution is iteratively *up*-sampled and cumulatively integrated with a weighted sum

\begin{equation*} { {\textit {CVM}} = \sum _{s} \widetilde { {\textit {CVM}} }_{s} },~ \text {with} \tag{11}\end{equation*}

$\widetilde { {\textit {CVM}} }_{s} = \widetilde {{\textit {CVM}}}_{s-1}^{{\textit {up}}} + \max \left ({\,\alpha _{s} ({\textit {CVM}}_{s} \cdot \epsilon _{s}), \widetilde {{\textit {CVM}}}_{s-1}^{{\textit {up}}} }\right)$, and $\epsilon _{s} = \max \left ({\,0\, {\textit {BDM}}_{s} \cdot (1-{\textit {BGM}}_{s}) }\right)$. Analogously, the tensor field ${\textit {TF}}$ is integrated in the Log-Euclidean domain as

\begin{equation*} { {\textit {TF}}_{({\textit {LE}})} = \frac {1}{{\textit {CVM}}} \sum _{s} \widetilde { {\textit {CVM}} }_{s} \cdot {\textit {TF}}_{s,({\textit {LE}})} }. \tag{12}\end{equation*}

The vesselness contributions are weighted here so that the resulting multi-resolution maximal filter response is balanced and equalized across scales. The boundary and background maps’ contributions in $\epsilon _{s}$ boost the spatial resolution of nearby tubular structures. The intensities of *CVM* can be further skewed towards high-, rather than low-, spatial frequency bands by modulating the gain $\alpha _{s}$. We adopt $\alpha _{s} = 1$ in the remainder of this paper. We also enforce the Euclidean ${\textit {TF}}$ to have unit determinant at each image voxel; the tensors’ magnitude, expressed by ${\textit {CVM}}$, is decoupled from the anisotropic and directional features throughout the whole multi-scale process. In this way, the synthesized and integrated *CVM* and *TF* maps constitute a consistent Riemannian vesselness potential.

### D. Exhaustive Geodesic Connectivity Paradigm

**Fig. 4.**
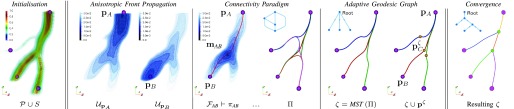
Exhaustive geodesic connectivity paradigm: topological inference of the over-connected graph $\sf \Pi $ and of its geodesic minimum spanning tree $\zeta $. *Vector* topology of the over-connected graph $\sf \Pi $ (first iteration), of its minimum spanning tree $\zeta $ (first iteration), and resulting tree topology at convergence.

Under the assumption that vessels join by minimal paths, and following the concepts introduced by Benmansour and Cohen [10], Kimmel and Sethian [34], Konukoglu *et al.* [36], [37], and Sethian [59], we present an anisotropic front propagation algorithm that, combined with an extended and exhaustive connectivity paradigm, joins multiple sources $\tilde {S} \mapsto S$ propagating on the Riemannian vesselness potential $\mathcal {P}$. Since we want to extract generic geodesic paths between points, we minimize an energy functional of the form $\mathcal {U}(\mathbf {x}) = \min _{\pi } \int _{\pi } \mathcal {P} \left ({\pi (\mathbf {x}), \pi '(\mathbf {x}) }\right) d \mathbf {x}$ for any possible path $\pi $ between two generic points along its geodesic length, so that $\| \nabla \mathcal {U} (\mathbf {x}) \| = 1$, and $\mathcal {U}(S) = 0$. The solution of the Eikonal equation is given here by the anisotropic fast marching algorithm, where front waves propagate from $S$ on $\mathcal {P}$, with $\mathcal {P}\left ({\pi, \pi ' }\right) = \sqrt {\pi '^{t} \cdot \mathcal {M} \cdot \pi ' }$ describing the infinitesimal distance along the path $\pi $, relative to the anisotropic tensor $\mathcal {M}$. In our case, $\mathcal {M} = {\textit {TF}}$, and $\pi ' \propto {\textit {CVM}}$. Note that this anisotropic level-set is a generalized version of the isotropic propagation medium, $\mathcal {M} \equiv I_{3}$. Together with the anisotropic fast marching, a non-greedy connectivity paradigm is run until convergence to extract the set of multiple minimal paths, which determines the over-connected geodesic graph $\Pi $ (Fig. 4).

#### 1) Initialization:

the set of binary seeds $\tilde {S}$ is first aligned towards the vessels’ mid-line with a constrained gradient descent on ${\textit {CVM}}$, so that the aligned individual seeds, together with the endpoints and branch-points of possible connected components, constitute the initial set of source points $\mathbf {p} \in S$, and initialize the anisotropic front propagation (Fig. 4).

#### 2) Connectivity Paradigm:

for any source point $\mathbf {p}$, propagating on $\mathcal {P}$, the geodesic energy map $\mathcal {U}_{\mathbf {p}}$ is iteratively computed and updated until complete exploration of the potential’s domain (or up to a pre-determined spatial neighborhood of $\mathbf {p}$ for computational efficiency), similarly to a front wave arrival-time map. We refer to [37] for further details of the individual fast marching step and for the implementation of the whole anisotropic front propagation algorithm. Each possible pair of source points, i.e. $\left ({\mathbf {p}_{A},\mathbf {p}_{B}}\right)$, is then connected with the geodesic minimal path $\pi _{{\textit {AB}}}$, by back-tracing the functional $\mathcal {F}_{{\textit {AB}}} = \left ({\mathcal {U}_{\mathbf {p}_{A}} + \mathcal {U}_{\mathbf {p}_{B}} }\right) + | \mathcal {U}_{\mathbf {p}_{A}} - \mathcal {U}_{\mathbf {p}_{B}} |$ from the source points $\mathbf {p}_{A}$ and $\mathbf {p}_{B}$ to the respective connecting geodesic point of minimal energy $\mathbf {m}_{{\textit {AB}}}$, identified as $\mathcal {F}_{{\textit {AB}}}\left ({\mathbf {m}_{{\textit {AB}}} }\right) = \min \left ({\mathcal {F}_{{\textit {AB}}} }\right)$ (Fig. 4). The connecting geodesic path $\pi _{{\textit {AB}}}$ is therefore obtained by the union of two geodesics, each of which is traced with a gradient descent on $\mathcal {F}_{{\textit {AB}}}$. The associated integral geodesic length $F_{\left ({\pi _{{\textit {AB}}} }\right)} = \int _{\mathbf {p}_{A}}^{\mathbf {p}_{B}} \mathcal {F}_{{\textit {AB}}}d\pi $ is determined along the extracted path $\pi _{{\textit {AB}}}$ and the connectivity of the graph $\Pi $ is accordingly updated. Here, $\Pi $ can be directly expressed as a canonical undirected weighted graph $\Pi = \left ({N,E }\right)$ comprising a set of *nodes* $N$, i.e. the set of points $\mathbf {p}$, and a set of *edges* $E$, i.e. the set of connecting paths $\pi $, respectively. By using a symmetric adjacency matrix, the integral geodesic length $F_{\left ({\pi _{{\textit {AB}}} }\right)}$ is then attributed to the edge’s weight, which connects the pair of nodes $\left ({\mathbf {p}_{A},\mathbf {p}_{B}}\right)$. It is clear that, by terminating the minimal paths’ extraction only with the initial set of source points $S$, the topology of the resulting geodesic connecting graph $\Pi $ would hinge on the initialisation, thus on the initial guess of the nodes, and would also constrain the connecting paths (i.e. the vascular branches) to connect (or bifurcate) only in correspondence of the initial set of source points in $S$.

#### 3) Adaptive Geodesic Graph:

with this view, we introduce an adaptive and self-organizing connectivity strategy for the geodesic graph $\Pi $, so that the topology of the graph itself will be plastically refined and updated in a completely automatic fashion. This is obtained by *i.* extracting the minimum spanning tree (MST) of $\Pi $, i.e. $\zeta = {\textit {MST}}\left ({\Pi }\right)$; *ii.* increasing the density of source points (nodes) at each connecting path (edge) of $\zeta $; *iii.* running the connectivity paradigm as in Section II-D2 among the new set of nodes and the existing ones. Note that the adaptive connectivity strategy employs an iterative process that will converge to a pre-defined spatial nodes’ density. In detail, the minimum spanning tree $\zeta $ is defined as the subset of the connected edges that acyclically links all the nodes together by minimizing the sum of total edge weights. Here, the edge weights are the integral geodesic lengths $F$, therefore the resulting $\zeta $ is the connected subset of geodesic minimal paths. Given, now its generic connecting path $\pi ^{\zeta }_{{\textit {AB}}}$, a new source point $\mathbf {p}_{C}^{\zeta }$ is generated between $\mathbf {p}_{A}^{\zeta }$ and $\mathbf {p}_{B}^{\zeta }$ so that $\mathbf {p}_{C}^{\zeta }$ is the respective midpoint of the geodesic path $\pi ^{\zeta }_{{\textit {AB}}}$, and

\begin{equation*} \| \mathbf {p}_{A}^{\zeta } - \mathbf {p}_{C}^{\zeta } \| \geq \mu,\quad \text {and}\quad \| \mathbf {p}_{B}^{\zeta } - \mathbf {p}_{C}^{\zeta } \| \geq \mu. \tag{13}\end{equation*}

$\mu $ is here the Euclidean spatial threshold for contiguous nodes and constitutes the pre-defined maximal spatial node density. The new set of source points $\mathbf {p}^{\zeta }$ will be connected with the existing ones following the connectivity paradigm as in Section II-D2, updating therefore the adjacency matrix that increases in size at each iteration. The process terminates when the pre-defined spatial nodes’ density is reached. Note that $\Pi $ is iteratively refined and the topology the associated MST may subsequently change from its initial guess (as in Fig. 4, first iteration vs. convergence). Also, the smaller $\mu $, the more dense the set of $\mathbf {p}^{\zeta }$, the finer the localisation of branch-points, the greater the complexity of the over-connected graph $\Pi $.

### E. Vascular Minimum Spanning Tree

The resulting vascular tree $\zeta $ is finally determined as the minimum spanning tree of the over-connected graph $\Pi $, as in Section II-D3, at convergence. Note that for more complex vascular topologies, a set of minimum spanning trees (i.e. a forest of geodesic MSTs) can be extracted for the underlying anatomical tree-like structures under a specific region of interest (ROI), by means of a co-registered binary or multi-class fuzzy mask. Here, the integral Euclidean length $L_{\left ({\pi ^{\zeta } }\right)}$ and the aforementioned integral geodesic length $F_{\left ({\pi ^{\zeta } }\right)}$ of each connecting path $\pi ^{\zeta }$ can be employed to modulate the extension of the resulting vascular tree(s) $\zeta $. Undesired leaves and possible spurious branches detected by the exhaustive connectivity paradigm can be pruned using $L_{\left ({\pi ^{\zeta } }\right)}$ and $F_{\left ({\pi ^{\zeta } }\right)}$, respectively. Lastly, by identifying a root, the hierarchical topology of the undirected vascular tree(s) is automatically determined, and each node is assigned with an univocal *parent-child* relation.

## III. Datasets and Experiments

### A. Datasets

A collection of 10-images datasets of synthetic vascular trees ($128\times 128\times 128$ voxels, isotropic 1 mm^3^) was generated using VascuSynth [28] considering three levels of increasing noise and increasing terminal branches (Table I).

**TABLE I:** Synthetic Datasets of Vascular Trees Generated With [28]

Label	$\blacktriangledown $	Gaussian	Salt&Pepper	Shadows	$\vartriangle $	Leaves
${\textit {NL}}^{\vartriangle }_{\blacktriangledown }$	${a}$	$\mathcal {N}(0,15)$	0%	0	${I}$	20
	${b}$	$\mathcal {N}(0,50)$	2%	0	II	30
	${c}$	$\mathcal {N}(0,50)$	10%	1	III	40

Real clinical angiographies were also considered: 24 Rotational Angiographies of cerebral Aneurysms (*RAA*) from the Aneurysk^1^ dataset [3]; 18 cerebral time of flight MR Angiographies (*MRA*) from the Kitware^2^ dataset [14]; 10 head-neck Phase Contrast (*PC*) MR venograms; and 10 cerebral Computed Tomography Angiographies (*CTA*). Vascular network ground-truths (GT) or manual annotations Gold Standard (GS) are given as spatial centerlines.^1^http://ecm2.mathcs.emory.edu/aneuriskweb/index^2^https://data.kitware.com/#collections

### B. Experiments

#### 1) Vesselness’ Connectedness:

The vesselness responses of the considered images are determined using VTrails (VT). The connectedness of the synthesized scalar map is qualitatively assessed in Section IV and the tensor field (*TF*) is inspected for a representative subset of angiographies. For the synthetic datasets, the scalar vesselness responses are also determined using the classical Frangi filter (FFR)^3^ [25], the Optimally Oriented Flux (OOF)^4^ [39], the current state-of-the-art method by Ranking the Orientation Responses of Path Operators (RORPO)^5^ [47], and the noise-reduction anisotropic Hybrid Diffusion with Continuous Switch filter (HDCS) ^3^ [45].^3^Implementation: http://www.tubetk.org^4^Implementation: https://www.mathworks.com/matlabcentral/fileexchange/41612-optimally-oriented-flux–oof–for-3d-curvilinear-structure-detection^5^Implementation: http://path-openings.github.io/RORPO

The histogram of the scalar vesselness maps is analyzed at different noise levels foreground (fG) – i.e. the tubular structures – and background (bG) components are initially determined from components are initially determined from the uncorrupted images. The associated histogram overlap (fG${\cap }$bG) is quantified for the obtained scalar filter responses from each method. Similarly, the foregroud-background separation range (fG${\leftrightarrow }$bG) is determined as the absolute difference between the 90-percentile of the background intensities and the 10-percentile of the foreground ones. The foreground interquartile range (fG_IQR_) is determined as well as the index of the intensity spread for the enhanced tubular structures. Lastly, the correlation of the fG components with the uncorrupted images is evaluated with the Spearman correlation coefficients fG$_{\varrho }$. Significant differences of the considered methods against the proposed one are evaluated with a pairwise Wilcoxon signed rank test.

#### 2) Semi-Automatic Connectivity–Synthetic Trees:

The connectivity paradigm described in Section II-D is used to infer the connected topology of the synthetic trees given the different scalar vesselness maps by FFR, OOF, RORPO and HDCS each associated with an isotropic tensor field, and the Riemannian vesselness potential $\mathcal {P}$ determined with VTrails (VT). Here, only the complete set of endpoints of the synthetic trees is given as user-defined initialisation seed. The reconstructed acyclic topology is compared to the ground-truth (GT). The robustness to image degradation is evaluated in terms of geometrical accuracy (symmetric error $\varepsilon _{S}$ and average Hausdorff_95%_ distance $\varepsilon _{H}$) globally for the synthetic trees and locally for the corresponding branchpoints. Branchpoints detection is considered within a GT spatial neighborhood of 5 mm. Trees’ topology is compared against the GT using both the spatially-aware DIADEM^6^ metric [27], and the tree edit distance (TED)^7^ [53], where differences are evaluated in terms of branches and branchpoints spatial correspondence, and graph adjustments, i.e. node insertion and deletion. While the DIADEM metric is bounded by [0,1], 1 being the perfect match, the TED score has no upper bound. Low TED scores represent higher topological matching, however, to obtain comparable indices of trees’ overlap, we adopt

\begin{equation*} \text {TED}_{\text {ov}} = \left ({1-\dfrac {\text {TED}(\zeta _{1},\zeta _{2})}{\text {TED}(\zeta _{1},\left \lbrace{ }\right \rbrace) + \text {TED}(\zeta _{2},\left \lbrace{ }\right \rbrace)} }\right) \cdot 100 \,\%,\qquad \tag{14}\end{equation*}

where $\zeta _{1}$ and $\zeta _{2}$ are the trees to compare, and $\left \lbrace{ }\right \rbrace $ represents a void graph. TED_ov_ has the same bound [0,1], 1 being the perfect match for isomorphic trees.^6^http://diademchallenge.org/metric.html^7^http://tree-edit-distance.dbresearch.uni-salzburg.at

#### 3) Fully Automatic Connectivity–Synthetic Trees:

Similarly to Section III-B2, the connected topology of the synthetic trees is inferred with VTrails using a fully automatic pipeline (VT_auto_); the Riemannian vesselness is considered as connectivity potential, the initial seeds for the exhaustive geodesic connectivity paradigm are automatically determined as in Section II-B, and the exploration of the Riemannian potential is limited to a pre-defined spatial neighborhood of the initial seeds. Given the GT, the evaluation of the geometrical and topological accuracy follows the previous scheme.

#### 4) Fully Automatic Connectivity–Clinical Data:

Each clinical angiography is processed using VT_auto_ and the accuracy of the inferred connected vascular topology is evaluated by comparing the resulting minimum spanning tree(s) with the available GT or GS annotations. As in Section III-B2, the symmetric error $\varepsilon _{S}$ and the average Hausdorff_95%_ distance $\varepsilon _{H}$ are provided globally for the vascular trees, and locally for the corresponding branchpoints. Branchpoints detection is determined within the GT spatial neighborhood. The topological correspondence is evaluated using the DIADEM metric. *RAA* centerlines are obtained with the Vascular Modeling Toolkit (VMTK) [5]; *MRA* ground-truth trees are determined with TubeTK [8]; the gold standard for *PC* and *CTA* datasets is given by the centerlines of the manual lumen segmentation, obtained with a skeletonisation strategy [30]. Note that, for whole-brain vascular datasets, only the intra-cranial volume was considered for the topological inference, by means of a co-registered brain mask, from the brainstem up to the cortex. Also, possible cycles in the GS have been opportunely cut or removed by adopting a ROI-based, conservative and intensity-maximizing, minimum spanning tree(s) extraction of the complete GS connected graph. Accordingly with the underlying anatomical tree-like vasculature, the quantitative analysis has been performed for the deep brain arterial trees [31] branching from the Circle of Willis in both *MRA* and *CTA* datasets, whereas we focused on the connectivity patterns of the posterior venous sinus in the *PC* datasets. Note also that additional effort was required to harmonize the provided centerlines in the form of a canonical acyclic graph (tree, or forest of trees), where branchpoints corresponds to nodes and vascular branches to edges respectively, since the tree topology cannot be consistently evaluated otherwise. This was performed with an in-house split-merge-connect strategy similar to [32].

## IV. Results

### A. Vessel Connectedness

**Fig. 5.**
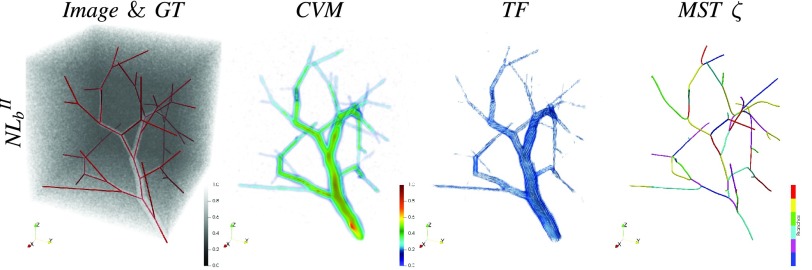
Representative example of synthetic tree using [28]: synthesized Riemannian vesselness and resulting minimum spanning tree with VT$_{\textsf {auto}}$.

**Fig. 6.**
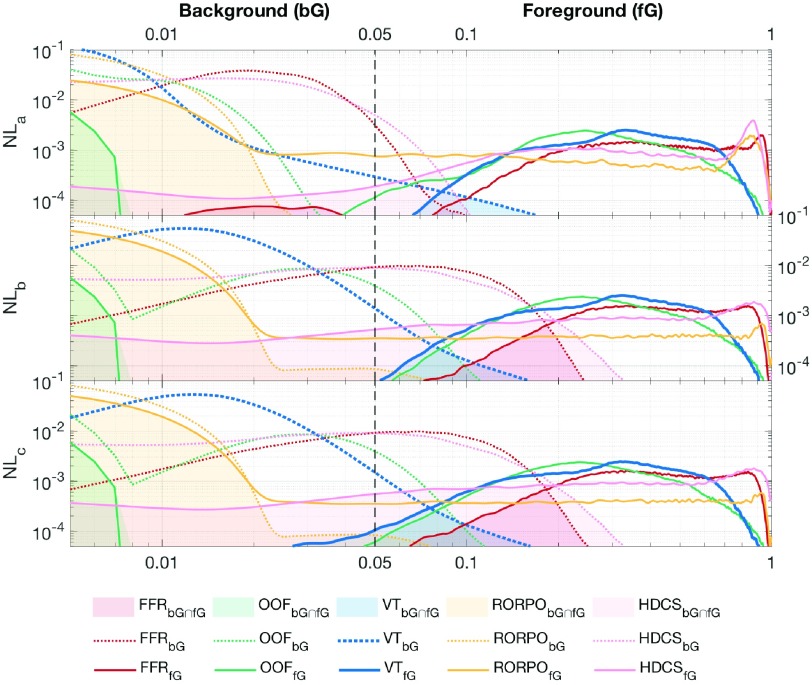
Histogram overlap for scalar vesselness with FFR, OOF, RORPO, HDCS and VT.

**Fig. 7.**
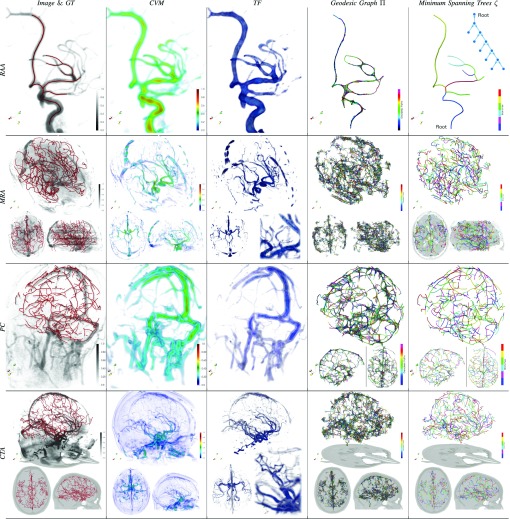
Representative set of clinical angiographies: synthesized Riemannian vesselness maps, geodesic graphs and resulting minimum spanning trees (VT$_{\textsf {auto}}$).

Figures 5 and 7 show the scalar and tensorial vesselness maps synthesized with VTrails (VT) for a representative subset of images. In all cases, VT strongly enhances the vessel connectivity, where low-resolution, noisy and fragmented (e.g. $NL_{b}^{II}$ and *PC*) vessels are recovered with a continuous and spatially smooth scalar filter response (*CVM*). High values and more defined local maxima are observed at structures’ mid-line, in correspondence of both regular and irregular tubular cross-sections, even in images with particularly degraded SNR, with improved noise rejection in the background. The connectedness of the vasculature is emphasized regardless the complexity of its shape, by spatially resolving nearby, tortuous and highly curvilinear vessels. In all synthetic and real clinical images, the synthesized tensor field (*TF*) shows consistent features with the scalar map and the intrinsic structure of the vasculature. Tensor orientation smoothly captures vessel directionality and higher anisotropy is found for the enhanced and connected vessels, whereas a predominant isotropic component is associated to the background. For the synthetic datasets, the scalar vesselness maps are also obtained with the Frangi Filter (FFR), the Optimally Oriented Flux (OOF), the state-of-the-art by Ranking the Orientation Responses of Path Operators (RORPO), and the noise-reduction anisotropic Hybrid Diffusion with Continuous Switch (HSCD) filter. The respective histograms are reported in Fig. 6, for the considered levels of increasing noise. After filtering, the discrimination of both foreground (fG), i.e. vessels, and background (bG) shows different trends for the considered enhancing methods (Table II). The area of histogram overlap (fG$\cap $bG), i.e. the confusion between fG and bG components, is lower in VT and FFR, compared to all other methods in all cases. For increasing noise, higher confusion between fG and bG is observed, with significantly higher ($p< 0.05$) histogram overlap values. Similarly, the separation of both fG- and bG-distribution tails (fG${\leftrightarrow }$bG) shows comparable values for FFR and VT with mild corrupting noise, whereas reduced values of fG${\leftrightarrow }$bG are observed for all FFR, OOF, RORPO and HDCS, with significantly worse separation ($p< 0.05$) at moderate-to-severe degradation levels. The intersection value of both fG and bG distributions is consistent in VT at different levels of corrupting noise, and lays in the vicinity of the ideal threshold (Fig. 6, black dashed-line). The foreground interquartile range (fG_IQR_) quantifies the smooth connectedness of the scalar filter response for the tubular structures, where a more compact and limited range suggests homogeneity and regularity of the scalar intensities in the neighborhood of enhanced structures. VT and OOF show comparable fG_IQR_ in terms of smooth filter-response connectedness, whereas significantly higher ($p< 0.05$) intensity ranges are found for FFR, RORPO and HDCS, suggesting increased variability or more distributed intensities for the filtered structures. High correlation coefficients (fG$_{\varrho }$) are found for HDCS, FFR and VT, where the intensities of the enhanced tubular-like structures monotonically correlate with the respective uncorrupted ones. In this case, HDCS has better performances ($p< 0.05$) for all noise levels, being the associated fG distribution rather skewed towards saturated hyper-intensities, in line with the intrinsic noise-reduction filter design.

**TABLE II:** Histogram Overlap (fG$\cap $bG), Foreground Vs. Background Separation Range (fG${\leftrightarrow }$bG), Foreground Interquartile Range (fG_IQR_), and Foreground Spearman Correlation (f$\text{G}_{\varrho}$) With the Uncorrupted Image, for FFR, OOF, RORPO, HDCS and VT Scalar Vesselness (Mean±SD). †: Significantly Worse (p < 0.05) and *: Significantly Better (p < 0.05) than VT in Paired Wilcoxon Signed-Rank Test

		FFR	OOF	RORPO	HDCS	VT
$NL_{a}$	fG${\cap }$bG	0.01±0.02	0.19±0.02^†^	0.23±0.07^†^	0.02±0.03	0.01±0.01
	fG${\leftrightarrow }$bG	0.21±0.04	0.01±0.01^†^	0.01±0.01^†^	0.14±0.03^†^	0.20±0.03
	fG_IQR_	0.44±0.03^†^	0.27±0.04	0.70±0.07^†^	0.51±0.04^†^	0.25±0.02
	fG$_{\varrho }$	0.78±0.05	0.70±0.04	0.70±0.14	0.87±0.07^*^	0.76±0.05
$NL_{b}$	fG${\cap }$bG	0.04±0.03	0.20±0.02^†^	0.51±0.05^†^	0.15±0.03^†^	0.01±0.01
	fG${\leftrightarrow }$bG	0.12±0.02^†^	0.05±0.01^†^	0.00±0.00^†^	0.03±0.01^†^	016±0.02
	fG_iQR_	0.39±0.02^†^	0.27±0.02	0.51±0.09^†^	0.48±0.03^†^	0.24±0.01
	fG$_{\varrho }$	0.76±0.05	0.70±0.04	0.66±0.09	080±006^*^	0.74±0.04
$NL_{c}$	fG$\cap$bG	0.05±0.02^†^	0.21±0.02^†^	0.52±0.03^†^	0.16±0.02^†^	0.02±0.01
	fG${\leftrightarrow }$bG	0.11±0.03^†^	0.05±0.01^†^	0.00±0.00^†^	0.02±0.01^†^	015±0.02
	fG_iQR_	0.39±0.02^†^	0.27±0.03	0.49±0.07^†^	0.48±0.03^†^	0.25±0.02
	fG$_{\varrho }$	0.75±0.05	0.69±0.05	0.66±0.06	079±006^*^	0.73±0.05

### B. Supervised Connectivity–Synthetic Trees

The accuracy of the reconstructed synthetic trees using different vesselness potentials is given in Table III for the whole trees’ geometry, for the detected branchpoints location and for the entire topologies. The symmetric errors ($\varepsilon _{S}^{\zeta }$) resulted overall comparable among the considered vesselness maps (FFR, OOF, RORPO, HDCS and VT), where a better performance (italic text) has been observed for VT (Table III.a). Slightly lower error distances are found on both $\varepsilon _{S}^{\zeta }$ and the distances ($\varepsilon _{H}^{\zeta }$) in all cases, being the former ones limited always within the voxel size. limited always within the voxel size. Above 80% of branchpoints were successfully detected in all cases, even with high level of corrupting noise. The considered enhancing methods yielded comparable values for the accuracy of the branchpoints’ location (Table III.b), however VT showed overall lower symmetric errors lower Hausdorff_95%_ distances ($\varepsilon _{H}^{\text {bp}}$), especially at high level of ($\varepsilon _{H}^{\text {bp}}$), especially at high level of degrading noise. This first suggests that the smooth Riemannian vesselness improves the accuracy of branchpoint spatial location, secondly, that the topological inference via the presented connectivity paradigm is considerably more stable even with different vesselness potentials. This is supported by the TED_ov_ indices (Table III.c), where considerable topological overlap is found for all the reconstructed trees. Better performances are observed for VT in the great majority of cases, especially for highly noisy images. DIADEM values show however that VT is outperforming all the other methods with the spatially-aware topological reconstruction of the synthetic trees, where the accuracy of the branchpoint spatial location and of the branches geometry is considered jointly with the hierarchical parent-child relation.

**TABLE III:** Synthetic Trees – Symmetric Error $\varepsilon_{S}$ [mm] and Distance $\varepsilon_{H}$ [mm] for the Minimum Spanning Trees ($\zeta$) Trees ($\zeta$) [$a$.], and for the Branchpoins (bp) Location [$b$.] (Mean±SD). Topological Accuracy [%]: $\textsf{TED}_{\textsf{ov}}$ and DIADEM [$c$.]

$a$.	$\varepsilon _{S}^{\zeta }$						$\varepsilon _{H}^{\zeta }$					
	FFR	OOF	RORPO	HDCS	VT	VT_auto_	FFR	OOF	RORPO	HDCS	VT	VT_auto_
$NL_{a}^{I}$	0.89±0.34	0.87±0.32	0.91±0.37	0.93±0.38	0.85±0.31	0.99±0.34	1.53±0.21	1.40±0.17	1.64±0.23	1.67±0.21	1.39±0.12	1.54±0.25
$NL_{a}^{II}$	0.93±0.32	0.93±0.32	0.95±0.33	0.99±0.40	0.90±0.31	1.03±0.34	1.48±0.05	1.46±0.07	1.57±0.12	1.79±0.26	1.39±0.07	1.57±0.07
$NL_{a}^{III}$	0.95±0.35	0.94±0.37	0.96±0.37	1.02±0.46	0.90±0.32	1.04±0.36	1.59±0.08	1.61±0.07	1.66±0.13	1.96±0.32	1.43±0.07	1.60±0.07
$NL_{b}^{I}$	0.91±0.34	0.88±0.30	0.98±0.44	0.96±0.43	0.84±0.30	1.03±0.47	1.53±0.12	1.37±0.15	1.83±0.28	1.82±0.31	1.34±0.19	1.93±1.30
$NL_{b}^{II}$	0.95±0.36	0.92±0.35	1.01±0.46	1.11±0.71	0.89±0.32	1.02±0.35	1.58±0.07	1.52±0.05	1.96±0.35	2.65±1.70	1.42±0.04	1.55±0.06
$NL_{b}^{III}$	0.96±0.38	0.93±0.37	1.04±0.57	1.12±0.70	0.90±0.33	1.51±1.21	1.66±0.07	1.58±0.05	2.35±0.51	2.62±0.63	1.46±0.08	3.94±6.61
$NL_{c}^{I}$	0.88±0.35	0.85±0.33	0.95±0.44	0.95±0.47	0.82±0.31	1.12±0.71	1.50±0.17	1.45±0.15	1.85±0.26	1.75±0.54	1.35±0.19	2.43±1.78
$NL_{c}^{II}$	0.96±0.36	0.92±0.34	1.02±0.48	1.17±0.77	0.87±0.32	1.04±0.38	1.64±0.11	1.50±0.07	2.04±0.25	2.68±1.12	1.40±0.08	1.65±0.13
$NL_{c}^{III}$	0.96±0.36	0.95±0.36	1.07±0.57	1.25±0.91	0.90±0.33	1.05±0.39	1.61±0.06	1.59±0.06	2.34±0.31	3.35±2.01	1.44±0.06	1.67±0.11
$b$.	$\varepsilon _{S}^{\text {bp}}$						$\varepsilon _{H}^{\text {bp}}$					
	FFR	OOF	RORPO	HDCS	VT	VT_auto_	FFR	OOF	RORPO	HDCS	VT	VT_auto_
$NL_{a}^{I}$	2.25±1.09	2.18±0.95	2.19±1.02	2.19±0.97	1.96±0.96	1.91±0.78	4.36±0.41	4.13±0.60	4.08±0.77	4.04±0.77	3.74±0.46	3.61±0.63
$NL_{a}^{II}$	2.19±1.00	2.05±1.03	2.18±0.99	2.30±1.11	2.03±0.97	1.92±0.90	4.08±0.32	4.12±0.60	4.03±0.46	4.29±0.35	3.82±0.68	3.69±0.55
$NL_{a}^{III}$	2.38±1.02	2.42±1.06	2.39±1.09	2.41±1.12	2.06±1.07	2.06±0.92	4.24±0.36	4.38±0.27	4.32±0.45	4.54±0.31	4.18±0.46	3.84±0.35
$NL_{b}^{I}$	2.38±0.97	2.23±1.13	2.50±1.14	2.66±1.09	1.89±0.92	2.07±1.05	4.15±0.33	4.36±0.33	4.32±0.38	4.39±0.51	3.68±0.63	4.21±0.46
$NL_{b}^{II}$	2.25±0.97	2.35±1.05	2.19±1.08	2.47±1.04	2.01±0.97	2.02±0.90	4.09±0.42	4.29±0.35	4.38±0.41	4.13±0.77	3.93±0.52	3.70±0.55
$NL_{b}^{III}$	2.38±1.07	2.25±1.12	2.09±1.08	2.46±1.14	2.15±1.04	2.16±1.04	4.35±0.31	4.39±0.48	4.28±0.50	4.51±0.21	4.03±0.41	3.75±1.41
$NL_{c}^{I}$	2.35±1.04	2.31±1.12	2.28±1.09	2.36±1.00	2.12±1.16	2.08±0.91	4.05±0.56	4.38±0.68	4.31±0.31	3.79±0.69	4.43±0.49	3.69±0.59
$NL_{c}^{II}$	2.33±1.04	2.23±1.01	2.23±1.04	2.41±1.11	1.95±0.98	2.01±0.88	4.14±0.40	4.06±0.71	4.31±0.37	4.23±0.78	3.91±0.46	3.72±0.53
$NL_{c}^{III}$	2.35±1.04	2.35±1.08	2.11±1.09	2.47±1.02	2.08±1.06	2.08±0.98	4.15±0.36	4.28±0.54	4.19±0.40	4.13±0.51	3.92±0.47	3.91±0.58
$c$.	TED_ov_						DIADEM					
	FFR	OOF	RORPO	HDCS	VT	VT_auto_	FFR	OOF	RORPO	HDCS	VT	VT_auto_
$NL_{a}^{I}$	74.6±8.2	70.3±5.3	79.1±6.7	70.1±3.3	73.5±8.5	75.4±6.3	43.3±28.6	39.0±28.8	43.6±29.4	39.3±30.9	52.4±18.8	65.1±12.3
$NL_{a}^{II}$	78.5±4.5	74.7±5.0	72.8±6.2	60.9±6.7	78.0±5.4	75.9±5.1	26.8±27.5	39.4±20.0	27.8±24.5	25.3±32.2	42.6±26.9	67.8±6.6
$NL_{a}^{III}$	73.2±4.9	69.7±3.1	71.3±5.0	58.8±8.5	74.6±6.4	74.6±4.6	44.4±27.3	25.4±28.6	51.3±26.9	22.8±25.9	57.6±9.2	71.5±6.6
$NL_{b}^{I}$	77.6±5.3	71.8±4.5	72.2±4.4	62.4±5.9	78.4±6.5	68.4±10.0	36.6±30.0	37.2±32.5	30.2±28.4	26.8±30.1	49.7±25.9	58.5±13.7
$NL_{b}^{II}$	77.8±5.7	73.5±7.5	71.6±3.9	58.6±8.1	76.8±4.6	74.6±6.6	41.5±25.1	40.2±28.4	31.5±19.8	22.7±28.8	53.9±18.1	57.4±15.9
$NL_{b}^{III}$	72.9±5.6	68.8±4.7	68.9±3.9	67.1±5.9	73.4±4.6	58.8±9.2	39.7±29.5	31.2±27.6	39.1±26.3	32.2±25.9	60.8±12.3	44.1±20.1
$NL_{c}^{I}$	77.2±6.9	70.7±6.4	72.1±3.4	55.4±12.5	78.7±7.6	64.7±4.9	42.7±26.5	36.6±34.8	30.8±28.4	12.9±22.6	54.3±22.3	44.2±9.8
$NL_{c}^{II}$	74.7±7.6	72.0±7.5	73.8±3.2	60.2±9.3	74.3±3.5	71.6±7.6	45.2±26.2	32.6±26.3	50.4±19.0	26.9±23.3	51.9±25.2	57.8±5.8
$NL_{c}^{III}$	75.6±3.3	69.5±4.6	69.5±3.0	65.1±3.7	74.8±4.0	68.4±4.4	36.1±24.3	18.9±27.2	24.0±27.3	23.4±26.9	47.7±21.1	43.8±20.9

### C. Fully Automatic Connectivity–Synthetic Trees

The reconstruction of the synthetic trees is performed in a completely automatic fashion, using VTrails with a fixed seeds quantile threshold ($Q_{p = 75\%}$) and without performing further pruning. The same aforementioned accuracy indices are reported in Table III (VT_auto_). As few terminal branches were missing at higher levels of degrading noise, the global $\varepsilon _{S}^{\zeta }$ slightly increases compared to the semi-automatic VT pipeline, however errors are overall comparable to the voxel size in all cases. Smaller average symmetric errors and $\varepsilon _{H}^{\text {bp}}$ values are found for the detected branchpoints location, suggesting that the Riemannian vesselness potential, combined with the fully automatic seeds initialisation, accurately recovers the junction points of the network. Such configuration outperforms the semi-automatic approach even with severely degraded images. Similarly to the semi-automatic approaches, the isomorphic topological overlap (TED_ov_) shows comparable values; no significant differences were found in the pairwise comparison, whereas the spatially-aware DIADEM metric reported higher matching in the majority of cases with sporadic significantly better values ($p< 0.05$) for VT_auto_ vs. (i.e. ${NL_{a}^{II}}$, ${NL_{a}^{III}}$ and ${NL_{c}^{II}}$ in Table III).

### D. Fully Automatic Connectivity–Clinical Data

The fully automatic VTrails is employed to recover the vascular trees from real angiographies. The quantitative assessment of the clinical datasets focused on vascular branches originally defined and provided in the available GT and gold standard. Co-registered ROI-based masks were used to separate intra/extra-cranial vessels and anterior/posterior or left/right-lobe vascular territories, coherently with assumption of deep-brain vascular trees as described in Section III-B4. Similarly to Section IV-C, the seeds quantile threshold is fixed to $Q_{p = 75\%}$, and further automatic pruning is performed to the vascular trees on leaves (i.e. vascular terminal branches) up to 5 mm length. Both geometrical and topological accuracies are reported for each clinical dataset in Table IV, where only the DIADEM metric is considered for the evaluation of the tree topology. In this case, TED_ov_ is not used, since the evaluation of the isomorphic tree overlap is uninformative and possibly misleading in an experimental setup other than simulated and synthetic images. The average symmetric errors $\varepsilon _{S}^{\zeta }$ were comparable to the voxel size, with the average distances ($\varepsilon _{H}^{\zeta }$) that did not exceed 5 mm. Analogously the detected branchpoints reported detected branchpoints reported a mean error $\varepsilon _{S}^{\text {bp}}$ of approximately 2 mm, with maximal distances up to 4-5 mm in all clinical datasets. DIADEM metrics showed a considerably high correspondence between the available ground-truth and the automatically reconstructed tree topology, with overall consistent and comparable values among different imaging modalities. The spatial and topological correspondence can be also qualitatively assessed in Fig. 7, where representative examples are shown with associated ground-truths (GT), geodesic graphs ($\Pi $) and resulting geodesic minimum spanning trees ($\zeta $). A forest of geodesic MSTs has been extracted for whole brain images, where nodes spatially correspond to vessel junctions and connecting edges to vascular branches, respectively. This suggests that VTrails can automatically and accurately infer the cerebrovascular topology at different scales with a vectorial representation.

**TABLE IV:** Clinical Angiographies – Symmetric Error $\varepsilon_{S}$ [mm] and Hausdorff Distance $\varepsilon_{H}$ [mm] of Tree ($\zeta$) AND BRANCHPOINTS (bp) (Mean±SD) – Topological Tree Accuracy DIADEM[%]

	$\varepsilon _{S}^{\zeta }$	$\varepsilon _{H}^{\zeta }$	$\varepsilon _{S}^{\text {bp}}$	$\varepsilon _{H}^{\text {bp}}$	DIADEM
$RAA$	0.407±0.307	1.101±0.436	1.309±0.665	2.452±1.315	78.87±14.81
$MRA$	0.535±0.489	1.167±1.081	2.084±1.157	4.167±0.559	77.68±8.22
$PC$	1.761±1.427	4.983±2.94	2.505±0.949	3.749±1.023	77.47±10.88
$CTA$	0.88±0.648	2.266±1.143	2.087±1.021	3.966±0.883	85.59±9.56

## V. Discussion and Conclusions

In this work, we presented VTrails, an automatic connectivity-oriented method for 3D cerebrovascular imaging, able to infer the brain vessels topology in the form of over-connected geodesic graphs, whose minimum spanning trees underlie anatomical deep-brain vascular trees. By using SLoGS within a coherent mathematical framework, the simultaneous synthesis of both scalar and tensorial vesselness maps consistently embeds smoothly connected tubular responses together with the underlying vascular anisotropy and directionality. Contrary to [19] and [20], where tensors are derived from fitting the image data, our method has the advantage of generating high-order vascular maps with few curvilinear templates. The vesselness maps recovered with SLoGS resulted less sensitive to noise and artifacts, and did not require any further regularization or positive-definiteness constraint, since anisotropic tensors are well defined for the described smooth and compact Gaussian kernels. Results in Section IV-A and Section IV-B demonstrate the robustness of our method to different levels of corrupting noise. This mainly stands as a sanity test with regards to traditional and popular tubular ridge detectors and enhancement techniques [25], [39], [45], [47] in case of images with severely impaired SNR. Regarding the enhancement and reconstruction of tortuous and convoluted tubular structures, the multi-resolution scale factor and the seed points cardinality, as observed in [9], also play a critical role in our case. On the one hand, they allow for a fully automatic processing pipeline; on the other hand, they modulate the computational complexity of the filtering step and of the subsequent topological inference. The proposed fully automatic processing pipeline is meant to avoid time-consuming, non-reproducible and user-dependent initialisations that could bias the objective inference of the over-connected vascular graph. A full-scale-range analysis/synthesis of the multi-resolution image pyramid should account for vascular structures of different size. Also, a reasonable choice for the seed points cardinality, here expressed with a fixed seeds quantile threshold ($Q_{p = 75\%}$) as in Section IV-C and Section IV-D, should trade-off between the computational complexity and the informative content of the filter-response. From our experiments, we observed that a low quantile (i.e. high seeds cardinality) can severely increase the complexity of the filtering step, without introducing information to the resulting scalar and tensorial (*CVM* and *TF*) maps; whereas, a high-value quantile can reduce dramatically the complexity (and the computational time), in detriment of vascular details. In the Appendix, we further provide the empirical computational time for the presented experiments and the performance benchmarking associated to a convoluted hand-crafted phantom.

The advantage of the proposed anisotropic level-set combined with the connectivity paradigm in Section II-D consists in optimally exploring and locally refining the geodesic domain of connecting paths, which yields topologically self-organizing vascular graphs and the associated minimum spanning trees. In [10], a similar level-set formulation focused on the extraction of shortest paths joining individual (or multiple) pairs of endpoints, without, however, determining the connected topology among the same set of points. In Section IV-B, the reconstruction of the synthetic trees showed overall good and comparable results even by adopting different vesselness maps. We assume an anisotropic level-set as proposed in [10] would have similar accuracies to VTrails, by employing our self-organizing connectivity paradigm. To the best of our knowledge, this is the first work where the accuracy of an automatically reconstructed set of vascular trees from clinical multi-modal brain angiographies is evaluated within a spatially- and topologically-aware validation framework. In all clinical datasets, both geometrical errors of the geodesic paths and the associated topological similarity evaluated on the centerlines ground-truth demonstrate that VTrails is able to accurately recover the cerebrovascular network at different scales with a vectorial representation. The sub-voxel average accuracy reached by VTrails in the clinical datasets suggests that the proposed approach can support intra-operative sessions with a patient-specific model up to a pre-defined level of detail, where surgical guidance is required and/or mini-invasive vascular repair is feasible. In general, the assumption of a vascular tree provides a natural and anatomically valid model for 3D cerebrovascular images, with few exceptions, e.g. the complete Circle of Willis and rare macroscopic anastomoses observed in the posterior cerebral circulation [11], [31], [63], [64]. For this reason, we first focused on the quantitative analysis of major deep-brain arterial (or venous) vascular trees, e.g. the anterior/posterior and left/right arterial branches from the Circle of Willis in *MRA* and *CTA*, as reported in Section IV-D; we then regard Fig. 7 for a qualitative inspection of the remaining smaller portions. The image resolution of the clinical angiographies does not allow for the inference of capillaries in the cortex (where the anatomy is more prone to show cyclic structures [29]), also the lack of established quantitative metrics to assess and compare cyclic and fully-connected topologies impede to focus particularly on the evaluation of anastomoses, at this stage. With the development of standardized metrics for fully-connected networks comparison, along the lines of [18], [43], and [55], future works would account a more specific validation focusing on cyclic structures at different scales, since VTrails can fully capture and embed in the over-connected vascular graph all the possible anatomical and geodesic connecting redundancy in the form of multiple local cycles. Note that these may underlie even actual anatomical anastomoses (Fig. 7). It is important to note that VTrails’ minimum spanning tree extraction formulation does not enforce any cerebrovascular anatomical prior per se, however, extra vascular-related constraints and associated anatomical connected topologies can be included with a user-defined initialisation to correct for specific locations where the vascular network is not acyclic. In our case, we employed co-registered ROI-based territorial masks to coherently extract a forest of geodesic MSTs in whole-brain clinical angiographies, as described in Section II-E. The proposed framework is completely modular, therefore further priors can be introduced and injected at different levels. Recent studies of the venous vasculature in the brain [12], [48], [62] show the potential of combining multi-modal imaging to determine a multi-parametric venous atlas and composite segmentation from Susceptibility-Weighted Imaging (SWI) venography and Quantitative Susceptibility Maps (QSM). Although in the present contribution we did not employ any combined multi-modal angiography, the introduction of an anatomical prior derived by the aforementioned methods and imaging modalities could improve, in first instance, the separation of the arterial from the venous side in whole-brain images, up to a pre-defined detail. Then, it could constitute a valid ground to infer a vectorial representation of the complementary cerebral venous vasculature. Further analysis, in future works, would consider a more complete and multi-spectral vascular dataset, since here we first focused on the performance and accuracy of the proposed method in a range of clinical scenarios, where individual, sometimes noisy angiographies are available. In the considered clinical datasets, major deviations from the centerlines ground-truth were observed for small and terminal vessels, where the effect of the limited spatial resolution and image quality degradation is predominant. This suggests that the detection of capillaries and those tiny vessels not well spatially resolved in the image may require a more supervised processing pipeline. Also, different connectivity patterns are found with VTrails for smaller vessels at high depths of the arterial (or venous) vascular trees. As side note, the considered centerlines gold standard do not constitute an exhaustive and flawless topological reference, since mis-connections, missing branches and manual discontinuous annotations may be present in the datasets. As shown in Fig. 7, our manual annotations can be noisy, sometimes fragmented and rather prone to misclassification among a pool of experts, especially in case of low contrast-to-noise and low image resolution (*PC*). We also observed that bad gold standard annotations may penalize the accuracy metrics presented in Section IV-D. For this reason, we put particular effort with selecting and evaluating the manually annotated GS for both *CTA* and *PC* datasets. To the best of our possibilities, we performed the skeletonisation of the manually lumen segmentation, as well as the extraction of the territorial GS minimum spanning trees, by means of a conservative and intensity-maximizing tree-extraction strategy of the complete GS connected graph, minimizing at the same time, the irremediable number of cycles cuts at smaller scales. It can be observed that possible minor mis-classifications in the available ground-truths and gold standards, as well as those from VTrails, may considerably affect the topological similarity metric of vascular trees of different size. Despite the optimal formulation of the Riemannian vesselness potential in conjunction with the proposed connectivity paradigm, narrow and spatially close vessels may eventually produce a geodesic short-cut with VTrails.

**Fig. 8.**
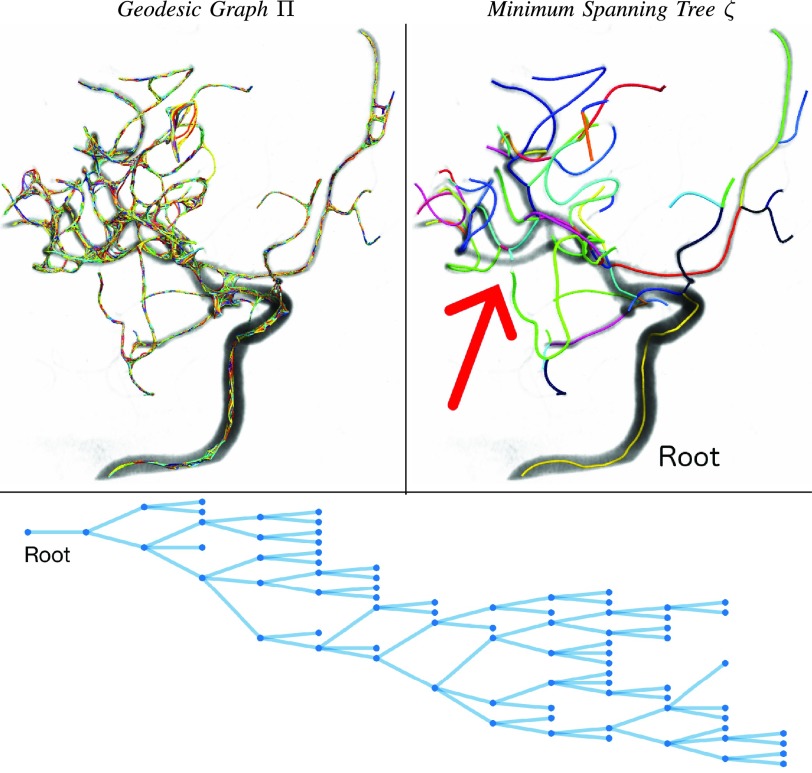
*RAA* topological inference with VT$_{\textsf {auto}}$. Geodesic Graph $\sf \Pi $ (left) and Minimum Spanning Tree $\zeta $ (right) of the underlying anatomical vascular tree. Missing branch in $\zeta $, due to a geodesic short-cut from *kissing-vessels*. Vectorial representation in the form of a connected hierarchical graph (below).

As shown in Fig. 8 for a randomly selected *RAA* image processed without restricting the topological inference, the extraction of the minimum spanning tree underlying the anatomical vascular tree can result in a missing branch (red arrow) due to a geodesic short-cut from *kissing-vessels*. Conversely, the over-connected geodesic graph encodes and preserves all the redundant connectivity. Although the *kissing-vessel* artifact may represent a potential limitation to the direct extraction of the minimum spanning trees, further improvements are being investigated to minimize its disruptive effect on the vascular network. In [50], we argue that minimum spanning tree(s) should optimally and robustly be extracted *after* the injection of population anatomical priors, propagated through pairwise or groupwise geodesic vascular graph alignment. With this view, such anatomical prior would compensate for biologically incompatible mis-connections and anatomically implausible geodesic short-cuts. Also, labeling priors would guide the extraction of the vascular trees towards their most anatomically meaningful realizations. Should the group-wise analysis of the vasculature determine a valid anatomical prior for extracting more accurate and refined subject-specific vascular graphs, at the same time, the novel *vectorial* approach could potentially impact on traditional vascular image analyses. Similarly to [60], by means of longitudinal and cross-sectional vascular graph-matching, registration and alignment, an over-complete graph of the cerebrovascular system could be determined with both arterial and venous components, and would ideally constitute a comprehensive, quantitative and data-driven vascular atlas of the human brain. This would support, in future works, a better understanding of the morphological and functional normality of the neurovascular system, also of the associated variability and pathology. The analysis and inference of clinically relevant biomarkers, such as vascular morphometric parameters (e.g. cross-sectional lumen boundaries, level of stenosis, aneurysm characterisation), functional markers (e.g. territorial supply, ischaemic events, local (de-)oxygenation, tracers wash-in/out), hemodynamic descriptors (e.g. blood flow, pressure, wall tension) and surrogate vascular indices (e.g. familial and environmental risk factors), could also be performed on a discrete, non-uniform and highly non-linear vectorial domain, which might be representative of a heterogeneous population. Given the encouraging results presented in this work, VTrails’ stands as first step towards multiple complimentary cerebrovascular applications, from supporting patient-specific interventional neuroradiology and vascular surgery, to population-wise studies of comparative neurology, neurovascular phylogenetics, and cerebrovascular disease progression on a larger scale.

## Figures and Tables

**Fig. 9.**
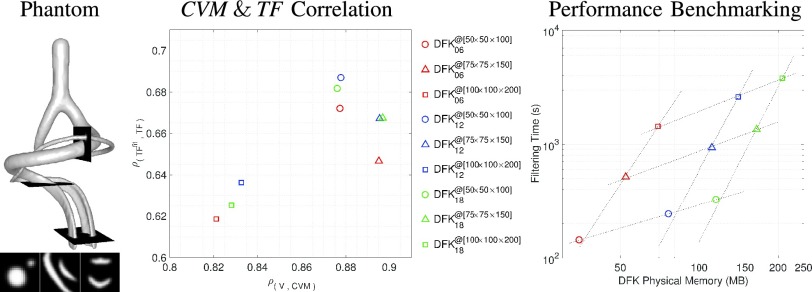
Parameters robustness and performance analysis on 3 DFKs and using a convoluted phantom of size $1.0\times $, $1.5\times $ and $2.0\times $ [$50\times 50\times 100$] voxels.

## Appendix Discrete DFK and Curvilinear Parameters

Finite SLoGS kernels are derived by opportunely sampling the 3D continuous impulse-response $\Gamma $ and the associated second-order derivative filtering kernel $K$. Analogously, the discrete ellipsoidal tensorial matrix $T$ is sampled in the Log-Euclidean space. In the present work we adopted templates of $5\times 5\times 5$ voxels for all the aforementioned instances. The adopted dictionary of filtering kernels (DFK) was generated as one-time configuration step prior to all filtering, and accounted for a total number of 12 different SLoGS (i.e. DFK = DFK_12_) of varying shape and curvilinear bending/tilting of the support. A complete list of parameters is detailed in Table V for each SLoGS in the DFK, along with the discrete degenerate scalar kernels $\delta {\textit {LoG}}$ and $\nu {\textit {LoG}}$.

**TABLE V:** SLoGS Parameters of the Adopted DFK

												
$\sigma _{1}$	5.0	5.5	6.0	5.0	5.0	5.5	6.0	6.0	6.0	6.0	5.5	5.5
$\sigma _{2}$	1.0	1.4	1.8	1.0	1.0	1.4	1.8	1.8	1.8	1.8	1.4	1.4
$\sigma _{3}$	1.0	1.0	1.0	1.0	1.5	1.3	1.1	1.3	1.2	1.2	1.0	1.0
c_0_	0.00	0.00	0.00	0.00	0.00	0.00	0.00	0.00	−0.60	0.60	0.40	−0.40
c_1_	0.00	0.10	0.20	0.00	0.00	0.10	0.20	0.20	0.20	0.20	0.10	0.10
c_2_	0.00	0.00	0.00	0.03	0.05	0.03	0.01	0.03	0.02	0.02	0.00	0.00

The discrete impulse-response ($\Gamma $) and derivative ($K$) degenerate kernels $\delta {\textit {LoG}}$ and $\nu {\textit {LoG}}$ are defined for a finite $3\times 3\times 3$ cubic template as

\begin{align*} \Gamma _{\delta {\textit {LoG}}} = \Big \lbrace \begin{smallmatrix} 1&~\text {for $\textbf {v}$}\,=\,[2,\,2,\,2]\, \\ 0&~\text {otherwise}\, \end{smallmatrix} \quad & K_{\delta {\textit {LoG}}} = \Big \lbrace \begin{smallmatrix} -\frac {26}{27}&~\text {for $\textbf {v}$}\,=\,[2,\,2,\,2]\, \\ \frac {1}{27}&~\text {otherwise}\, \end{smallmatrix}, \\ \Gamma _{\nu {\textit {LoG}}} = \begin{smallmatrix} \frac {1}{27}&~\forall \,{\textbf {v}}\, \end{smallmatrix} \quad & K_{\nu {\textit {LoG}}} = \begin{smallmatrix} \frac {1}{27}&~\forall \,{\textbf {v}} \end{smallmatrix},\end{align*}

with **v** the indexed voxel position within the cubic template.

Orientation Sampling in 3D

As described in Section II-B, the early tubular salinecy map $V_{{\textit {tube}}}$ is determined by means of an icosphere of subdivision level $n$ from which we initially sample orientations in 3D. $n$ is fixed and set equal to 2 for all the scales. This produces an initial number of 1080 different orthonormal bases, i.e. $\boldsymbol {\omega }_{\text {ico}}^{{\textit {all}}} \in \mathbb {R}^{3}$, which further reduces to 81 different orthonormal bases ($\boldsymbol {\omega }_{\text {ico}}^{{\textit {all}}} > \boldsymbol {\omega }_{\text {ico}}^{{\textit {reduced}}} = \boldsymbol {\omega }_{\text {ico}} \in \Omega _{\text {ico}}$) when the fully symmetric $K_{{\textit {tube}}}$ kernel is employed as in (5).

The subsequent image filtering formulated in (7) and (10) employs the whole SLoGS DFK and focuses on data-driven orientations $\boldsymbol {\theta } \in \Theta $ identified by the seeds $\tilde {S}$ (see Section II-B). The seeds-related orientations $\boldsymbol {\theta }$ may change in number with respect to $\boldsymbol {\omega }_{\text {ico}} \in \Omega _{\text {ico}}$. In our implementation, the seeds-related orientations $\boldsymbol {\theta } \in \Theta $ can differ from each other by a *maximum* in-plane angle of $\frac {\pi }{12}$, evaluated on both azimuth- and elevation-angle planes for all the orthonormal bases components.

### Robustness of SLoGS Parameters

The analysis of the robustness of SLoGS parameters is perfomed here by considering the original DFK = DFK_12_, and other 2 similar dictionaries of different cardinality, i.e. DFK_6_ and DFK_18_. The evaluation of the filter response of the considered dictionaries accounts for the voxel-wise Pearson correlation between the original image and the resulting scalar filter response (*CVM*), i.e. $\varrho (V, {\textit {CVM}})$, and the voxel-wise Pearson correlation of the tensor field (*TF*) directionality and anisotropy with the tensor gold standard in the Log-Euclidean space, i.e. $\varrho ({\textit {TF}}^{\text {fit}}, {\textit {TF}})$. Performance benchmarking was also performed in terms of DFKs physical memory load and empirical computational time. For the evaluation, a convoluted hand-crafted phantom presented in [49] was employed at different size and resolution (Fig. 9).

The correlation values of the synthesized maps with the respective ground-truths are shown in Fig. 9. Since no publicly available ground-truth for direction and anisotropy exists, we necessarily derived the gold standard ${\textit {TF}}^{\text {fit}}$ by fitting the tensor field over the original phantom $V$. Similarly to (4), we enforced positive definiteness of the ellipsoidal matrix, by considering the absolute value of the image-based Hessian eigenvalues. Overall, similar and comparable correlations were observed for the considered DFKs, by processing the phantom at different image size. This suggested reproducible results and overall good robustness of the DFKs by adopting similar varying parameters. Clusters of values ranged between 0.82 ~ 0.90 and 0.62 ~ 0.69 for $\varrho (V, {\textit {CVM}})$ and $\varrho ({\textit {TF}}^{\text {fit}}, {\textit {TF}})$, respectively. A slight decrease of the linear correlations was found for DFK_6_. Following this trend, we assume a further reduction of the dictionary cardinality may result in poor tensorial vesselness maps.

### Computational Cost and Implementation

As observed in Section II-B and in Section V, the complexity of the framework hinges on the density (or sparsity) of different tubular structures in the image and on the desired level of vascular detail. A performance analysis is shown in Fig. 9 for the aforementioned set of DFKs combined with a phantom at multiple image size. Both filtering time and physical memory load of the DFKs reported an underlying power-law trend (dashed lines in Fig. 9) in the adopted implementation. The estimated range of memory load was 30 ~ 200 MB, and a maximum filtering time of approximately 1 hour was observed for the complete DFK_18_ combined with the most dense and detailed phantom. Heuristically, for a representative experiment on clinical angiography – whole-brain isotropic 1 mm^3^ (approximately $200\times 200\times 150$ voxels), we observed an average processing time of 2 ~ 5 hours with the adopted DFK = DFK_12_. This includes the full-scale-range analysis/synthesis of both scalar and tensorial maps, together with the exhaustive connectivity paradigm, accounting for an exploration neighborhood of 10 ~ 25 mm diameter.

We justify the choice of DFK as trade-off between the correlation indices previously observed, and the overall computational performance. In future works, the use of a DFK with no less than 6 SLoGS may produce similarly accurate results, minimizing the computational load. Although we used a high performance computing cluster to process all images in our experiments, the whole framework was implemented and tested in Matlab, single 3.1 GHz Intel Core i7. The code is available at https://vtrails.github.io/VTrailsToolkit/ as an open-source tool-kit.
